# Circularity of Smart Products and Textiles Containing Flexible Electronics: Challenges, Opportunities, and Future Directions

**DOI:** 10.3390/s25061787

**Published:** 2025-03-13

**Authors:** Ewa Skrzetuska, Paulina Rzeźniczak

**Affiliations:** Faculty of Material Technologies and Textile Design, Textile Institute, Lodz University of Technology, 116, Zeromskiego Str., 90-924 Lodz, Poland; paulina.szablewska@dokt.p.lodz.pl

**Keywords:** textronic, circularity, sustainable development, reuse, smart textiles, recycling

## Abstract

The integration of flexible electronics into textiles and smart products has revolutionized industries, enabling innovations such as wearable health monitors, interactive clothing, and energy-harvesting fabrics. However, the rapid growth of these technologies poses significant challenges for sustainability and circularity. This paper explores the concept of circular economy in the context of smart textiles and products containing flexible electronics. It highlights the technical, environmental, and economic challenges associated with their end-of-life management and proposes strategies to enhance circularity, including design for disassembly, advanced recycling technologies, and policy frameworks. The paper concludes with a discussion of future research directions to achieve a sustainable lifecycle for these advanced materials.

## 1. Introduction

Circularity in the textile sector is a key element of sustainable development strategy, aimed at reducing the impact of the clothing industry on the environment. The modern textile industry is characterized by a high consumption of raw materials and a significant generation of waste. Traditional production methods are based on the model of a linear economy, which leads to the excessive exploitation of natural resources. The introduction of a circular economy strategy in the textile industry aims to minimize waste and maximize the reuse of raw materials. A particularly significant challenge is the management of waste containing flexible electronics, which is a new segment in the textile sector [1,2,3].

The convergence of textiles and electronics has given rise to a new generation of smart products that combine functionality, connectivity, and wearability. Flexible electronics, including sensors, conductive polymers, and energy storage devices, are seamlessly integrated into textiles to create innovative applications in healthcare, sports, fashion, and beyond. However, the environmental impact of these products, particularly at their end-of-life, has raised concerns. Traditional linear economic models, characterized by a “take-make-dispose” approach, are ill-suited to handle the complexity of smart textiles and flexible electronics. A transition to a circular economy, which emphasizes reuse, recycling, and resource efficiency, is essential to mitigate the environmental footprint of these technologies [4,5,6].

The beginning of the 21st century is characterized by a global increase in the consumption of raw materials. By 2019, this parameter had increased by 66 percent, tripling since the 1970s and reaching 95.1 billion metric tons [7,8]. Around the world, about 50 million tons of electronic waste, also called e-waste, are produced every year. In 2022, it was predicted that 57 tons of this waste would be produced; this number amounted to a record 62 million, and considering the trend, by 2030, this number is expected to increase by another 20 tons. This aspect is becoming a huge problem related to recycling and caring for the environment [9,10]. With the growing presence of electronics, awareness of proper recycling is not growing. Most electronic waste, such as televisions, computers, phones, refrigerators, washing machines and others, is disposed of incorrectly and leads to the release of hazardous substances into the environment, including mercury, lead and cadmium [11]. The second-largest contributor to the overall percentage of waste is textiles. It is estimated that around 92 million tons of this type of waste are created annually. Unfortunately, this number is constantly growing, and this is due to the trend of “fast fashion”, which means a short lifespan of clothing. In the absence of preventive measures and the beginning of active recycling, the amount of waste could increase by as much as 60% by 2030 [9]. Currently, only 25% of textile waste is recycled. The rest ends up in landfills [12]. The most dangerous group of raw materials, from which textronic solutions are also produced, is polymers. It is estimated that every year, as much as 400 million tons of polymer waste—plastic—is created in the world. There are many representatives of this group, including polyethylene, polypropylene, polyester, PVC and many other plastics. They are used in all areas of life and especially in the production of everyday articles, electronics, clothing. Less than 10% of plastics are recycled [13,14]. Growing public awareness and initiatives aimed at reducing plastic production, recycling, introducing alternative solutions and limiting the negative impact on the environment are extremely important. The negative impact of the disposal, and mainly improper disposal, of the above-mentioned raw materials causes huge damage to the environment. It is necessary to specify this for heavy metals, such as silver or copper, and polymers, which cause water pollution, toxicity to all organisms, and the emission of toxic substances into the air. Graphene is also characterized by high chemical stability. It is difficult to decompose in natural conditions and can persist in the ecosystem for a long time. Additionally, the chemical changes are notable. Various forms of graphene, e.g., oxide graphene, can enter chemical reactions and pollute water and soil by releasing toxic compounds [15,16,17,18].

**Figure 1:**
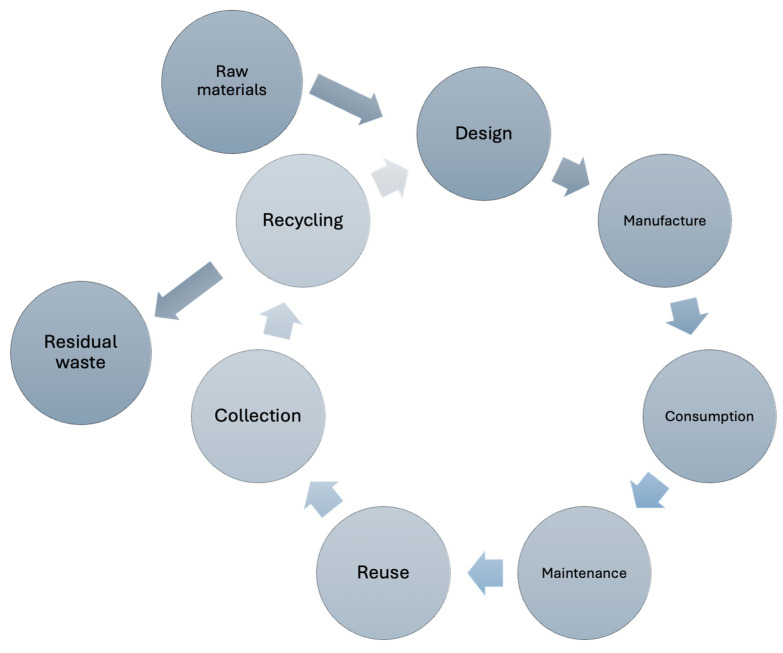
Circular economy (CE).

A circular economy (CE), presented in Figure 1, is a model of production and consumption that involves minimizing waste through reusing, repairing, refurbishing, and recycling existing materials and products. In the context of textiles and textile materials containing flexible electronics, a CE is becoming increasingly important due to the growing demand for sustainable solutions in the textile and electronics industry [19].

This paper examines the challenges and opportunities associated with the circularity of smart products and textiles containing flexible electronics. It also explores emerging solutions and outlines a roadmap for achieving sustainable lifecycle management.

## 2. Smart Textiles and Flexible Electronics

Smart textiles, also known as e-textiles or electronic textiles, represent a transformative convergence of textiles and electronics, enabling the creation of fabrics with enhanced functionality. By integrating flexible electronics into textiles, these materials can sense, react, and adapt to environmental stimuli, opening new possibilities in healthcare, sports, fashion, and beyond. The integration of these technologies is driving innovation, but it also raises important questions about sustainability, durability, and scalability.

Smart textiles are fabrics that incorporate electronic components to provide added functionality, such as sensing, communication, or energy-harvesting. These textiles are enabled by flexible electronics, which are lightweight, bendable, and stretchable electronic devices that can be seamlessly integrated into fabrics. The combination of textiles and electronics has given rise to a wide range of applications, from wearable health monitors to interactive clothing and energy-efficient buildings.

**Figure 2:**
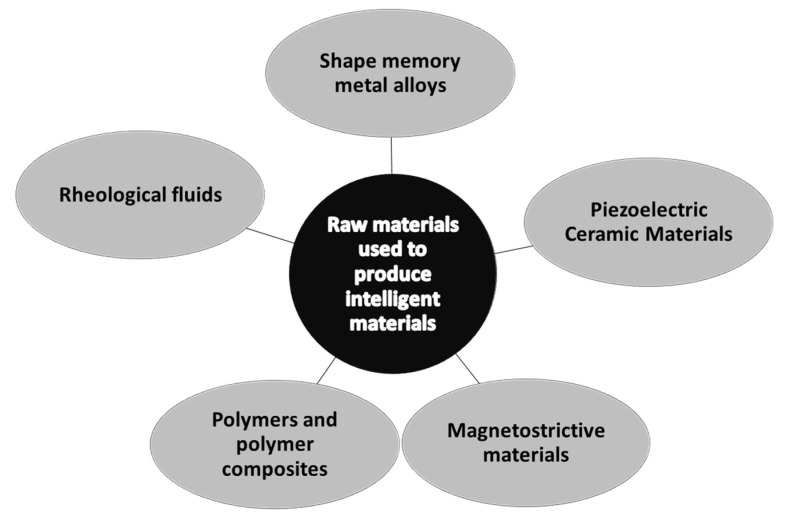
Division of intelligent materials according to the raw material used to produce them.

The development of intelligent textiles requires the incorporation of specific active materials into fiber materials or the modification of the surface of textile products to give them the intended properties. The development of a method for integrating interactive functions directly into the surface of the textile fibers, to construct a multi-interface response that provides a theoretical basis and strategic support for the design of the structure, will improve the performance and sensing mechanisms of novel intelligent wearable e-textiles. This will enable us to obtain intelligent products without the need for rigid and uncomfortable metal elements. Such solutions will enable the combination of special and sensory functions while ensuring the appropriate comfort of use of the textiles described. An intelligent material can respond to external stimuli by significantly changing its properties to respond to the given stimuli in a desired and effective way in a predictable manner. An intelligent–textronic material must function as a sensor, processor and actuator and demonstrate a real-time feedback effect. We can divide intelligent materials according to the raw material used to produce them. This is presented graphically in Figure 2 [20].

### 2.1. Key Components of Smart Textiles Systems

Conductive materials are the foundation of smart textiles, enabling electrical energy to flow through the fabric and facilitating functions such as sensing, heating, data transmission, and energy harvesting. The development of conductive materials has greatly advanced the field of e-textiles, improving their durability, flexibility, and integration with traditional textile structures. Based on the existing literature, conductive materials in smart textiles can be broadly divided into three main groups: metals, conductive polymers, and carbon-based materials.

Metals are widely used in smart textiles due to their excellent electrical conductivity and reliability. Silver is one of the most used metals in smart textiles due to its high electrical conductivity and antibacterial properties. Silver can be incorporated into fabrics in the form of coatings, nanoparticle dispersions, or conductive threads. Studies suggest that silver-coated fibers and silver nanoparticles increase conductivity while maintaining flexibility [21]. Copper is another highly conductive metal often used in smart textiles due to its cost-effectiveness compared to silver. However, its susceptibility to oxidation can reduce long-term performance. To prevent this, copper is often coated with protective layers or embedded in composite materials [22]. Gold is used in high-performance applications due to its excellent oxidation resistance and stable conductivity. Although more expensive than silver or copper, gold-coated fibers and nanolayers are used in medicine and high-end wearable electronics [23]. Metals can be integrated into textiles as conductive yarns and printed circuits, or they can be deposited as thin films using methods such as electroplating, vacuum deposition, or inkjet printing [24].

Conducting polymers provide a lightweight and flexible alternative to metals, making them particularly attractive for wearable applications. Key conductive polymers include PEDOT:PSS (poly(3,4-ethylenedioxythiophene)polystyrene sulfonate), which is widely used due to its high electrical conductivity, transparency, and processability in water-based solutions. Studies indicate that PEDOT:PSS-coated textiles exhibit stable electrical properties even under mechanical deformation [25]. Polyaniline is known for its tunable conductivity and chemical stability. It can be deposited on textile substrates via in situ polymerization or dip-coating, providing good electrochemical performance in sensor and energy storage applications [26]. Polypyrrole has been studied for its biocompatibility and stable conductivity. It is often used in biomedical smart textiles such as bioelectrodes and wearable biosensors [27]. Conducting polymers are advantageous due to their inherent flexibility and compatibility with textile structures, although challenges remain, such as stability under environmental conditions.

Carbon-based conductive materials have gained significant attention due to their exceptional electrical, mechanical, and thermal properties. The main types include graphene. This is a two-dimensional material with exceptional electrical conductivity and mechanical strength. It can be incorporated into smart textiles via solution processing, inkjet printing, or chemical vapor deposition. Graphene-based textiles have been developed for applications in wearable sensors, flexible energy storage, and electromagnetic shielding [28]. Carbon nanotubes offer high aspect ratios and excellent conductivity, making them suitable for stretchable and flexible conductive fabrics. Studies show that CNT-coated textiles exhibit excellent electromechanical properties and durability for use in smart clothing [29]. Carbon fibers, although mainly used for structural reinforcement, also have good conductivity and are used in hybrid conductive textile composites. Carbon-based materials are often used in combination with polymers or metals to improve electrical properties while maintaining flexibility and wear resistance. Sensors embedded in textiles can detect various environmental or physiological parameters, such as temperature, pressure, humidity, and biometric data (e.g., heart rate, muscle activity).

The integration of conductive materials into textiles has revolutionized the development of smart textiles, enabling a wide range of applications, from healthcare monitoring to interactive fashion. While metals provide high conductivity, conductive polymers and carbon-based materials provide flexibility and processability. Research continues to focus on improving the durability, washability, and scalability of these materials to make smart textiles more commercially viable.

Sensors embedded in textiles can detect various environmental or physiological parameters, such as temperature, pressure, humidity, and biometric data (e.g., heart rate, muscle activity). Smart textiles often require integrated energy sources or storage systems, including flexible batteries: thin, bendable batteries that power electronic components; supercapacitors: devices that store and release energy quickly; and energy-harvesting systems: technologies such as solar cells or piezoelectric materials that generate energy from the environment. Flexible circuits and interconnects enable communication between electronic components. These are often printed or woven into the fabric using conductive inks or threads.

### 2.2. Fabrication Techniques for Smart Textiles

The development of smart textiles relies on advanced fabrication techniques that integrate conductive materials into fabric structures while maintaining flexibility, durability, and functionality. Various approaches are used to embed electronic components within textiles, each with its own advantages and challenges.

Printing and coating methods involve applying conductive materials, such as silver nanoparticle inks or conductive polymers (e.g., PEDOT:PSS), onto textile surfaces. These methods allow for the precise patterning of electronic circuits while maintaining textile flexibility.

Screen Printing: A well-established technique in textile manufacturing, screen printing enables the deposition of thick conductive layers, making it suitable for large-scale production. Research by Stoppa highlights its effectiveness in producing robust, stretchable conductive traces [30].

Inkjet Printing: Inkjet technology offers the high-resolution deposition of conductive inks onto textiles, enabling the rapid prototyping of flexible electronics. This technique is particularly useful for producing intricate circuit designs with minimal material waste [31].

Spray Coating: Spray-coating techniques allow for the uniform deposition of conductive materials over large areas. Studies have demonstrated the use of graphene-based inks in spray-coated smart textiles, providing excellent conductivity and mechanical durability [32].

Traditional textile manufacturing methods, such as weaving and knitting, have been adapted for the integration of conductive yarns and fibers, allowing for the fabrication of electronic textiles with inherent flexibility and durability.

Weaving: Conductive fibers, such as silver-coated nylon or stainless-steel threads, can be interlaced with traditional fibers to form a durable, integrated circuit within the fabric [5].

Knitting: Knitted structures provide additional stretchability and flexibility, making them ideal for wearable sensors and biometric monitoring applications [18].

Embroidery techniques involve stitching conductive threads onto textiles to create customized circuits and decorative electronic patterns. Studies have shown that embroidered conductive pathways maintain mechanical stability even after multiple wash cycles, making them suitable for durable smart textiles [17]. Hybrid embroidery methods, combining conductive thread with traditional stitches, enhance both electrical performance and aesthetic appeal [33].

Lamination and encapsulation to improve the durability of smart textiles. Electronic components are often laminated or encapsulated within protective textile layers. Thin-film encapsulation techniques protect electronic circuits from moisture and mechanical wear, enhancing their longevity [34]. Conductive layers are embedded between fabric layers using polymer coatings, such as polyurethane or PDMS, to shield them from environmental factors [18].

Each fabrication technique offers unique benefits depending on the intended application of the smart textile. Printing and coating allow for high-precision electronic integration, while weaving, knitting, and embroidery provide structural flexibility. Lamination and encapsulation further enhance durability, ensuring that smart textiles can withstand real-world conditions. By leveraging these advanced fabrication methods, researchers continue to push the boundaries of wearable technology and functional textiles.

### 2.3. Applications of Smart Textiles

Intelligent textiles have found their application in a whole range of fields, from automotive to medicine. There are solutions that are useful in professional conditions for specialists and every day for citizens those that improve processes, increase safety or save lives, and there are those that are intended solely to improve the visual effect of a given product.

Starting with monitoring vital signs, wearable health monitors can track vital signs such as heart rate [35], respiration [36,37,38] and body temperature [39,40,41].

Textronic solutions are used as therapeutic devices. Fabrics for this purpose can deliver heat [42,43], compression [44] or electrical stimulation for medical treatments [45]. Systems have even been created for the rehabilitation of people after stroke [46].

The new thing is smart dressing. In medicine, the proper use of textronics could lead to the development of smart dressings that monitor the condition of wounds and provide information on necessary interventions [47,48].

In addition to the use of textronic solutions on adults, there is considerable research underway on products for children and even infants [49,50].

Moving on to sports and fitness, performance monitoring is key. Apparel used for this purpose tracks sports performance parameters such as muscle activity [51,52] or pulse measurements [53]. This also includes safety equipment such as smart helmets [54] or vests that monitor impacts and provide real-time feedback.

Textronics is also used in fashion and lifestyle. There are solutions such as interactive clothing. They can change color [55,56], display patterns [57] or respond to external stimuli [58]. There is also heated clothing such as jackets or gloves with integrated heating elements [59].

The latest technologies are mainly introduced to the military. They have several applications and are responsible for collecting data and processing it in many areas of defense. One such area is monitoring soldiers’ vital signs [60], improving their comfort and increasing safety when exposed to difficult weather conditions [61]. Textronics also supports camouflage. These are textiles that adapt to environmental conditions to increase concealment. Communication systems are also used in the military. In terms of intelligent solutions, these are uniforms with integrated antennas or communication devices.

Energy-harvesting fabrics are also starting to become available. These textiles generate electricity from sunlight or movement [62]. Textronics is also making its way into smart buildings, specifically into curtains or wall coverings that regulate temperature or lighting. The technology of changing the transparency of materials under the influence of electrical pulses is in the research phase and is not yet widely available on the market.

## 3. Challenges to Circularity

The circular economy in the textile industry faces numerous challenges resulting from the complexity of the materials used. Modern textiles, especially textronics, often consist of many components with different physicochemical properties, which significantly impedes their effective recycling.

In an era of growing environmental awareness and the need to minimize electronic and textile waste, the circular economy is a key challenge for the development of textronic products [63]. Textronics, combining advanced electronic technologies with flexible and durable textiles, finds application in many areas, such as smart clothing, healthcare and sports [64]. However, due to the complexity of the material structure and the presence of electronic components, recycling and reusing these products poses a serious technological and logistical challenge [65].

These components are often tightly bonded, woven, or coated with protective layers, making them difficult to separate. In addition, a strong adhesion of components is necessary to ensure product durability and longevity, but it also reduces the feasibility of disassembly at the end of the product life cycle.

In addition, high-performance textiles are designed to withstand harsh conditions, such as extreme temperatures, moisture, and mechanical stress. While this durability extends the product lifespan, it also makes the degradation and disintegration of materials more difficult when trying to recycle them. The need for specialized techniques to separate and recover valuable components without damaging their properties remains a significant barrier to achieving circularity in textile production.

### 3.1. Difficulties in Recycling and Reuse

Material complexity: Textronic products are composed of a variety of materials, such as fabrics, cables and electronic components, which are tightly integrated with each other. This integration makes it difficult to separate and recycle the individual components. Presence of hazardous substances: Electronic components may contain substances that are harmful to the environment, which complicates the safe processing and disposal of these products.

### 3.2. Lack of Standards and Regulations

Inadequate legal framework: Current regulations often do not consider the specificity of textronic products, leading to legal loopholes and hindering the implementation of effective circular practices. Need for new standards: There is a need to develop design and manufacturing standards that facilitate subsequent recycling and minimize environmental impact.

### 3.3. Technological Challenges

Advanced manufacturing processes: The production of textronic products requires complex technologies that are not always adapted to easy disassembly and recycling. Limited component durability: Electronic components may have a shorter lifespan than the textiles themselves, leading to premature product wear and waste generation.

### 3.4. Consumer and Producer Awareness

Low environmental awareness: Both consumers and producers are often unaware of the need to handle textronic products responsibly at the end of their life.

Lack of return and collection systems: Poorly developed collection systems for used textronic products make it difficult to reuse or recycle them.

**Figure 3:**
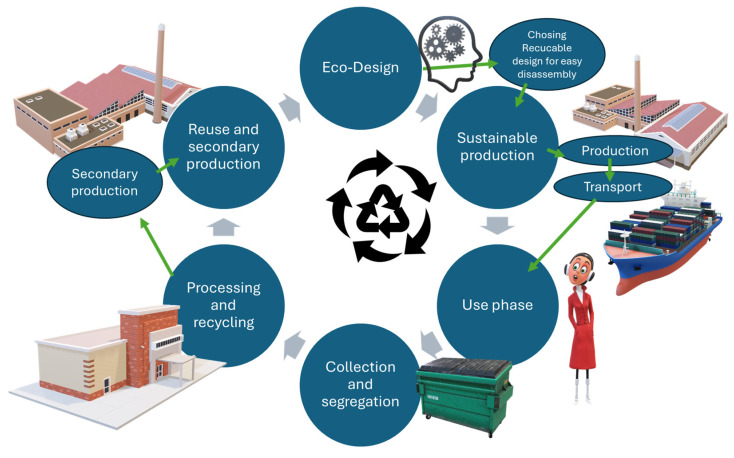
Block diagram of the closed loop of textronic products.

The closed loop of textronic products requires the effective management of raw materials, the development of new dismantling technologies and the implementation of appropriate legal regulations. Key aspects of this process include ecodesign, the effective separation and processing of materials and raising environmental awareness of consumers and producers (Figure 3). This document presents a flowchart of the closed loop of textronic products, considering key stages of the product life cycle and recycling and reuse strategies to minimize negative impacts on the environment.

The flow chart presented in Figure 3 shows the closed loop of textronic products:

1. Ecodesign—use of sustainable materials with high recyclability and modular design, enabling easy disassembly and elimination of harmful substances [66].
2. Sustainable production—implementation of technologies that reduce the consumption of raw materials and energy and reduce production waste [67].
3. Use phase—extending the product life through a repair, maintenance and reuse strategy [68].
4. Collection and segregation—effective recovery logistics including the identification, classification and separation of textile materials from electronic components [69].
5. Processing and recycling—mechanical and chemical methods of the recovery of textile and electronic raw materials, such as fiber separation, recovery of rare metals and biodegradation of selected components [70].
6. Reuse and secondary production—use of recovered materials in the new generation of textronic products, closing the raw material cycle and reducing the consumption of primary resources [71].

This diagram illustrates the closed life cycle of textronic products, supporting the principles of the circular economy and sustainable development by optimizing production, use and disposal processes.

### 3.5. Regulatory Challenges

The textile industry faces many regulatory challenges that make it difficult to implement circular economy principles. Smart textiles and flexible electronics are inherently heterogeneous, combining organic and inorganic materials with very different properties. This complexity complicates recycling and recovery processes, as materials need to be separated and processed individually. There are regulatory and legal challenges involved in implementing a circular economy for smart textiles.

### 3.6. Lack of Uniform Legal Regulations

Currently, there are no coherent and comprehensive regulations governing the recycling and disposal of smart textiles and flexible electronics. Different countries have different standards for handling electronic and textile waste, which makes it difficult to harmonize recycling processes at the international level [72]. The lack of clear regulations leads to a situation in which manufacturers and companies involved in the recovery of raw materials do not have guidelines on the methods of processing and segregation of these materials.

### 3.7. Classification of Waste and Difficulties in Their Categorization

Smart textiles are a unique category of products, combining the features of textiles and electronics, which causes problems related to their classification in legal systems [73]. Many waste management regulations lack a dedicated category for textronics, which means that these products are either wrongly classified as textile waste (which does not include electronic components) or as e-waste, despite their structure being different from traditional electronic devices. As a result, their disposal and the obligations of producers are unclear.

### 3.8. Lack of Producer Responsibility for the Entire Product Life Cycle

The implementation of extended producer responsibility (EPR) in the smart textiles sector is limited by the lack of dedicated regulations [74]. In many cases, producers are not obliged to ensure the recyclability of their products or to take back used products. Implementing EPR systems could force companies to design products in accordance with ecodesign principles and to provide infrastructure for recycling their products.

### 3.9. Chemical Regulation Constraints

Many smart textiles contain chemicals used to improve the conductivity, flexibility, or durability of materials, which may be subject to restrictive regulations, such as the Registration, Evaluation, Authorization, and Restriction of Chemicals (REACH) Regulation in force in the European Union [3]. Restrictions on the use of certain substances can hinder the production of more sustainable materials if alternative raw materials are not available that perform the same functions.

The first challenge in recycling is insulation electronic components. First, such components must be separate from textile materials. Textiles can have various elements such wires, detectors, batteries and displays. It is especially important to remove electronics to avoid the contamination of other materials. Items such as accumulators and batteries require separate recycling because they contain chemicals that are harmful to the environment if not disposed of properly. Many other electronic components also contain hazardous materials, such as heavy metals or toxic chemicals, which pose environmental and health risks if not properly managed at end-of-life. They should be properly disposed of and taken to collection points.

### 3.10. Material Traceability and Standardization Issues

Recycling regulations rely on the effective identification and segregation of materials. In the case of smart textiles, there is a lack of standardized labeling that would allow for the easy recognition of the product’s ingredients and their potential recyclability [75]. The absence of standardized design guidelines, labeling systems, and recycling protocols for smart textiles creates barriers to efficient end-of-life management. This results in a lack of adequate recycling infrastructure. There are still no developed recycling systems that would be adapted to recover both electronic components and textile materials in one process. The introduction of a product labeling system in terms of their composition and recyclability could significantly improve waste treatment processes and help manufacturers meet environmental standards.

### 3.11. Lack of Financial Incentives and Legislative Support

Recycling companies face financial difficulties related to the costs of separating and recovering raw materials from smart textiles, and the lack of appropriate subsidy programs or tax breaks makes investing in new technologies unprofitable [76]. Regulatory support, e.g., through the introduction of tax breaks for companies investing in green solutions, could accelerate the development of innovative processing technologies.

The smart textile industry faces serious regulatory and legal challenges that slow down the implementation of the principles of the circular economy. The lack of consistent legal standards, difficulties with product classification, limited producer liability and insufficient legislative support mean that recycling these materials remains a challenge. It is necessary to introduce clear regulations that consider the specificity of textronics, create a material traceability system and develop financial support mechanisms that will enable the effective implementation of solutions consistent with the circular economy.

## 4. Strategies for Enhancing Circularity

A circular strategy for textronic products must encompass the entire product life cycle—from design to production, use, disposal and material recovery. Key elements of circularity in this industry are sustainable design, reuse, recycling materials, innovative logistics and production processes. The authors of this article decided to prepare a detailed description of such an approach based on available data.

### 4.1. Design for Circularity

Design for textronic products must be sustainable and allow for easy decomposition of the product at the end of its life cycle. Key principles are:

- Selection of materials that are biodegradable, easy to recycle or reuse (e.g., recycled organic fabrics).
- Modular design—electronic components and fabrics must be easy to separate at the end of the product life.
- Minimization of the use of toxic chemicals and difficult-to-recycle materials (e.g., PCBs, difficult-to-separate synthetic fibers).

Pangaia uses recycled and biodegradable materials in its smart textiles. An example is their range of clothing made from organic fabrics and special fibers obtained from algae, which biodegrade at the end of the product life cycle. Additionally, they are working with technology companies to create clothing equipped with sensors that monitor the user’s health, with the possibility of easily separating the electronic components [4].

**Figure 4:**
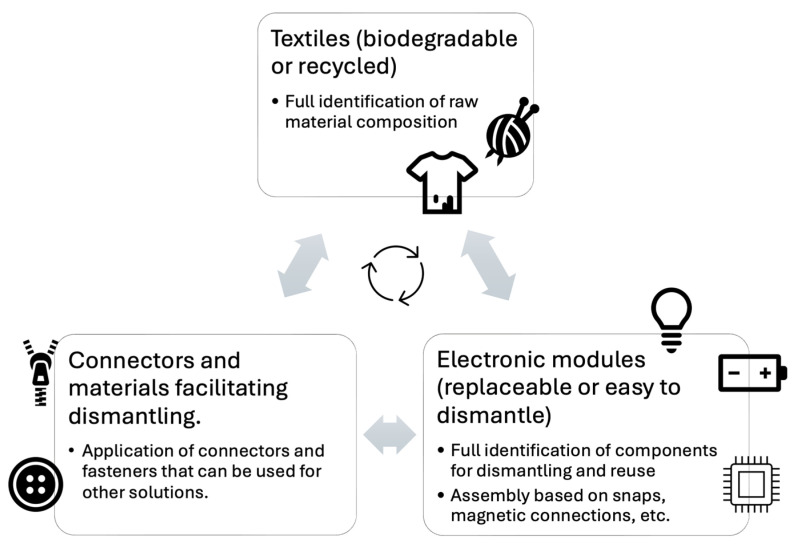
Graph: Modular design.

This graph in Figure 4 shows the modular design of a product, where different components (e.g., electronics, textiles, sensors) are designed to be easily separated during recycling. The graph highlights the following:

- Textiles (biodegradable or recycled).
- Electronic modules (replaceable or easy to disassemble).
- Connectors and materials that facilitate disassembly.

Product layout shows the separation of modules into sensors, batteries, and textiles. After separating the electronic components from textile material, they can be subjected to classic recycling, which involves recovering metals such as gold, silver or copper and other valuable materials from components such as integrated circuits, transducers or sensors. Textile materials can be subjected to traditional textile recycling methods such as upcycling and mechanical recycling. Upcycling consists of reusing materials to create new products, such as clothing, accessories or construction materials. Mechanical recycling involves processing textile waste into fibers that can be used to produce new fabrics.

E-textile return systems—creating programs encouraging consumers to return used smart textiles to manufacturers.

### 4.2. Sustainable Production

The production of textronic products should be based on the efficient use of resources. This means, among other things, the following:

- Optimizing production processes to minimize waste.
- Using recycled materials (e.g., recycled fabrics).
- Using renewable energy in production processes.

In cooperation with Parley for the Oceans, Adidas produces shoes and clothing from materials recycled from ocean plastic waste. The introduction of this technology reduces the amount of plastic used by 70%, while reducing the carbon footprint by about 40%. Textronic clothing based on this method could contain flexible recycled polyester fibers that can integrate electronics without affecting the strength of the material [4].

**Figure 5:**
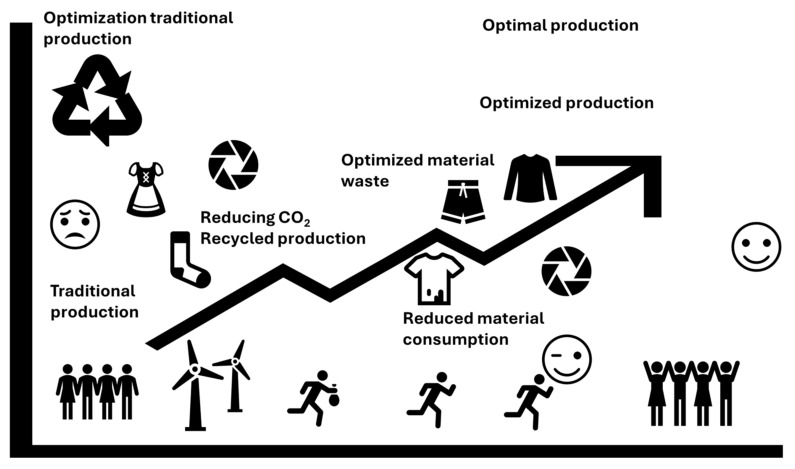
Optimizing the production process.

Figure 5 shows how optimizing production processes reduces CO_2_ emissions, waste and water consumption [58]:

- Reducing the use of primary materials (by 40–70% in the case of plastic).
- Using renewable energy in the production process (e.g., solar and wind energy).

A diagram with a production timeline shows the reduction of emissions and resources in successive stages. It compares traditional production processes with production optimized for recycling and renewable energy.

### 4.3. Use Phase

In this phase, it is crucial to extend the useful life of textronic products and promote consumer practices aimed at minimizing waste:

- Creating products with a longer lifespan that can be repaired, updated, upgraded (e.g., replaceable electronic modules).
- Educating users on how to care for textronic products to extend their lifespan.
- Innovative business models, such as the rental of textronic clothing, repair services, or return and recycling programs.

Patagonia offers its customers the possibility of returning used products that are repaired or recycled. The introduction of such a model in the textronic context could include service programs for smart clothing (e.g., replacement of used batteries, upgrade of sensors), which would extend their life cycle by 30–50% [4,58].

A very important aspect is also determining the product’s lifespan. This is helpful in the context of waste management and the possibility of creating a recycling process for this type of product. An example would be smart clothing from Hexoskin [77]. Their shirts have built-in sensors that collect information about vital signs such as heart rate, respiratory rate, activity, and body posture. The manufacturer states that the battery life of the clothing is 300 charging cycles, with a single charge lasting up to 36 h. The clothing itself is made of high-quality materials and will last for several years if used properly [78]. Despite the emerging textronic innovations, manufacturers are not obliged to specify this parameter in product specification [79,80].

### 4.4. End-of-Life Management

Managing end-of-life textronic products is a key element of a circular strategy. Important actions include the following:

- Creating an infrastructure for collecting used products, such as collection points in stores.
- Efficiently recycling materials such as textiles and electronic components and processing them into secondary raw materials.
- Developing technologies to separate electronic components from textiles without losing their value.

Fairphone builds smartphones based on the principles of modular design and recycling. Each component of the phone can be easily replaced, repaired or recycled. A similar approach could be used in textronics: clothes with replaceable electronic components that can be easily disassembled and recycled.

Table 1 presents the material recovery process, including recovery methods and examples of reuse. Table 2 presents a comparison of recycling methods for textronic products.

Effective reverse logistics is the foundation for circularity in textronic products. This requires strong supply chains and collaboration with partners at local and global levels.

- Expanding collection networks for end-of-life products.
- Introducing incentives for customers to recycle products (e.g., discounts on new products).

H&M’s “Take Back” program allows people to return used clothes in exchange for discounts on new products. In a textronic context, this could include returning used clothes with technology (e.g., smart shoes that track activity), allowing for the recovery and reuse of materials.

**Figure 6:**
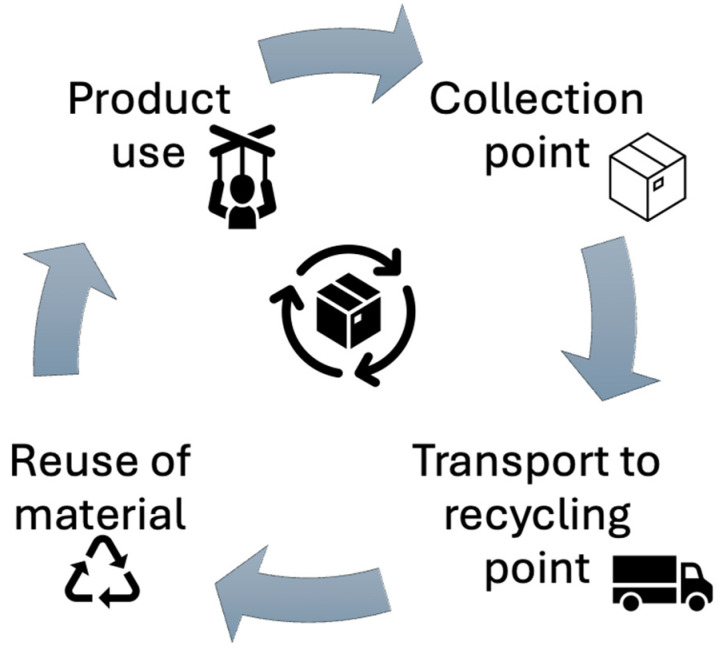
Reverse logistics.

Figure 6 it shows the flow of returned products from consumers through collection points, including transport to recycling plants and finally the reuse of materials in the production process.

Smart textiles are inherently difficult to recycle due to their hybrid nature. They combine traditional textile fibers (e.g., polyester, cotton, or nylon) with electronic components such as conductive threads, batteries, and microchips. These materials are often tightly integrated, making separation and recovery a daunting task. Additionally, the presence of hazardous substances in electronics, such as heavy metals and rare earth elements, complicates the recycling process. To address these challenges, two primary recycling methods are being explored: mechanical recycling and chemical recycling. Each method has its advantages and limitations, and their applicability to textronics depends on the specific materials and design of the smart textile. Mechanical recycling involves the physical processing of used fabrics so that they can be reused to produce new materials. This process consists of several stages. Initially, used textiles must be collected and sorted. Here, the division based on the type of material dominates, whether it is natural, artificial or synthetic. Quality must also be considered. Then, it is necessary to move on to shredding, where the materials are shredded into smaller fragments, to be subsequently subjected to the defibering process to obtain single fibers. The raw material obtained in this way must be cleaned, and any additives, such as buttons or zippers, must be separated. Now, new products can be created, i.e., the final stage—spinning and weaving. The disadvantages of this recycling are the possibility of destroying the fiber, or it may be shortened and/or weakened, which affects the quality of the final product. The method of mixing new fibers with those from recycling is very often used. In textronic products, more attention should be paid to the second and third stages, in which the textronic elements must be separated from the textile base [81,82].

Advantages:

1. Simplicity and Low Cost: Mechanical recycling is a well-established process with relatively low operational costs.
2. Scalability: It can handle large volumes of textile waste, making it suitable for mass-market applications.
3. Energy Efficiency: Compared to chemical recycling, mechanical recycling consumes less energy.

Limitations:

1. Material Degradation: Repeated shredding and processing weaken the fibers, reducing their quality and limiting their reuse in high-performance applications.
2. Inability to Separate Components: Mechanical methods struggle to separate electronic components from textile fibers, leading to contamination and loss of valuable materials.
3. Limited Applicability to Textronics: The presence of electronics often renders mechanical recycling ineffective, as it cannot recover functional electronic materials.

Mechanical recycling is best suited for smart textiles with minimal electronic integration or those designed for easy disassembly. For example, textiles with detachable electronic modules could allow the textile portion to be mechanically recycled while the electronics are processed separately.

Chemical recycling involves breaking down materials into their original chemical components so that they can be used to produce new fibers. This recycling method allows for obtaining high-purity raw materials. Currently, there are three methods of chemical recycling: hydrolysis, glycolysis, and pyrolysis. Hydrolysis deals with the decomposition of polyester in the presence of water. Thanks to this, terephthalic acid and ethylene glycol can be obtained. Glycolysis involves the reaction of polyester with an excess of ethylene glycol. This process leads to the production of oligomers. The last method is pyrolysis—the process of thermal decomposition of organic materials at high temperature without access to oxygen. This leads to the formation of oils, gases and solid residues [83,84]. An example is the technology developed in Germany to depolymerize polyesters used in textronics, which allows them to be reused in the production of new materials. It is important to continue to work on effective recycling methods for both traditional textiles and advanced textronic products to minimize their environmental impact.

Advantages:

1. High-Quality Output: Chemical recycling can produce virgin-quality materials, making it suitable for high-performance applications.
2. Separation of Materials: It can effectively separate blended materials, including textiles and electronics, enabling the recovery of valuable metals and polymers.
3. Versatility: Chemical recycling can handle complex materials, including those with embedded electronics.

Limitations:

1. High Cost: The process is expensive due to the need for specialized equipment and chemicals.
2. Environmental Impact: Some chemical recycling methods produce hazardous byproducts, requiring careful management to minimize environmental harm.
3. Energy Intensity: The process often requires significant energy input, reducing its overall sustainability.

Chemical recycling holds great promise for textronics, particularly for recovering precious metals from electronic components and regenerating high-quality fibers. For example, a smart jacket with integrated sensors could be chemically processed to recover copper from the conductive threads and regenerate polyester fibers for new textiles.

The global textile recycling market is expanding rapidly, driven by increasing awareness of environmental issues and regulatory pressures to reduce textile waste. According to recent estimates, the market is expected to grow at a compound annual growth rate (CAGR) of over 5% in the coming years. However, the recycling of smart textiles remains a niche segment, with few companies specializing in textronics recycling. Such companies include Worn Again Technologies, a UK-based company focused on chemical recycling of textiles, including blended and complex materials [85], and Circ, a US-based company that uses chemical processes to recycle polyester and cotton blends, with potential applications in smart textiles [86]. While these companies are making strides in textile recycling, the recycling of textronics is still in its infancy. Most existing solutions focus on traditional textiles, and the development of specialized processes for smart textiles will require significant investment and innovation.

A key question to ask yourself is: Is textile recycling profitable? The profitability of textile recycling depends on several factors, including the type of materials being recycled, the scale of operations, and the availability of markets for recycled products. Traditional textile recycling can be profitable, particularly for high-volume materials like polyester. However, the recycling of smart textiles is currently less economically viable due to the complexity and cost of processing [87].

The key challenges are the separation and recovery of electronic components add significant costs to the recycling process. The market for recycled smart textiles is still developing, with few buyers willing to pay a premium for recycled materials. The presence of hazardous materials in electronics may subject textronics recycling to stringent regulations, increasing compliance costs.

The opportunity of recycling is the recovery of precious metals and rare earth elements from electronic components could offset processing costs.

Designing smart textiles for easier disassembly and recycling could reduce processing costs and improve profitability. The recycling of smart textiles containing flexible electronics is a complex but essential endeavor. While mechanical and chemical recycling methods offer potential solutions, their effectiveness depends on the design and composition of the textronics. Chemical recycling holds promise for recovering high-quality materials and separating electronic components, but it requires significant investment and innovation to become economically viable.

The textile recycling market is growing, but the recycling of textronics remains a niche area with few specialized players. For the industry to thrive, collaboration between textile manufacturers, electronics companies, and recyclers will be essential. Additionally, designing smart textiles with end-of-life considerations in mind can facilitate recycling and improve profitability. As the demand for sustainable solutions continues to rise, the development of effective recycling technologies for textronics will play a critical role in shaping the future of smart textiles. By addressing the technical, economic, and regulatory challenges, the industry can unlock the full potential of textronics while minimizing its environmental impact [88].

## 5. Future Directions

To achieve full circularity in the production and use of smart textiles and flexible electronics, it is necessary to intensify research on several key aspects. Here are some directions that should be considered in future actions:

The development of standardized guidelines for design, labeling and recycling: The standardization of the design and labeling processes of textronic products is the foundation that will enable better and more organized recycling. Products must be designed in such a way that each of their components (textiles, electronics, sensors) can be easily separated and recycled. The guidelines should also include uniform rules for labeling materials that are clear to consumers and recyclers. This will allow for quick identification of recyclable components and their correct processing.

Example: A textile company could use QR codes on labels that provide quick access to information on the material composition and its recycling possibilities. The consumer, by scanning the code, learns how to return the product to the appropriate collection point.

Investing in scalable recycling technologies for smart textiles: Current recycling technologies are not fully adapted to advanced materials that combine textiles with electronics. In the future, efficient and cost-effective processes are needed to recover valuable materials, such as precious metals, from electronic components, and to process synthetic polymer fibers. These technologies should be scalable enough to sustainably process large volumes of textronic products.

Example: Research laboratories can develop methods to chemically separate textiles from electronic components, allowing both to be recycled in the same process. Such technologies would be used in specialized recycling plants that could handle large batches of clothing and accessories.

Promoting cross-sector collaboration between manufacturers, recyclers, and policymakers: Collaboration between different sectors is essential for circularity to work effectively. Manufacturers need to work with recyclers to optimize material recovery processes. At the same time, policymakers and regulators should introduce appropriate regulations that promote sustainable production and recycling. An example of such cooperation may be the joint creation of standards for materials and production processes that facilitate their subsequent recycling.

Example: The creation of a common cooperation platform where manufacturers and recyclers can exchange data on materials and recycling technologies, as well as develop more effective ways of returning products by consumers.

The exploration of bio-based and biodegradable materials for electronics: An important direction of research is the development of materials based on renewable raw materials, which are also biodegradable. Currently, electronics used in textronics are based on synthetic materials and metals that are difficult to recycle. Work on biodegradable electronic components, e.g., from conductive polymers, can significantly reduce the negative impact of textronics on the environment.

Example: Scientists are working on the development of biodegradable sensors and circuits that can be fully compostable at the end of the product’s life cycle. An example is the development of electronics based on plant fibers and organic polymers.

**Figure 7:**
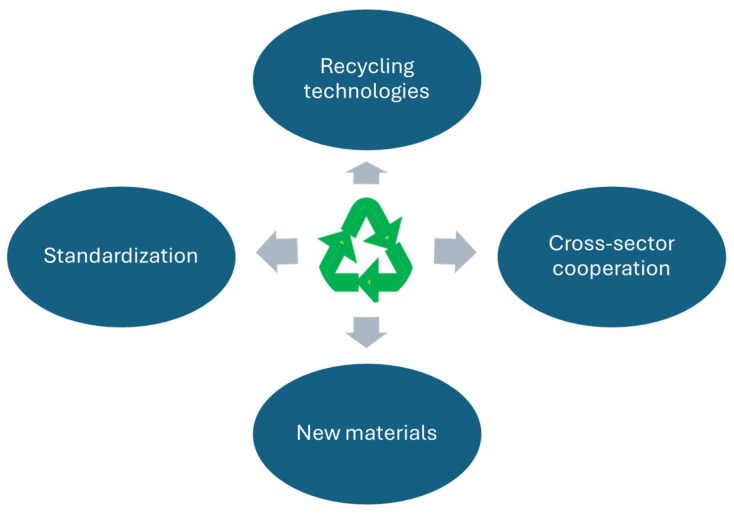
Future directions for circularity smart textiles and flexible electronics.

The framework in Figure 7 illustrates the future directions of action in the context of the closed loop of smart textiles and flexible electronics. It includes key areas such as standardization, recycling technologies, cross-sectoral cooperation and the development of biodegradable materials. The framework emphasizes the importance of cooperation and innovation in each of these areas.

The framework consists of the following elements:
Standardization:

- Guidelines for designers and manufacturers.
- Unified labeling rules (e.g., QR codes on textile labels).
- Requirements for the recyclability of products.

Recycling technologies:

- Development of recycling technologies for hybrid materials.
- Efficiency in material recovery.
- Scalability of processes.

Cross-sectoral cooperation:

- Coordination between manufacturers, recyclers and regulators.
- Standards and regulations supporting recycling and sustainable production.
- Common platforms for the exchange of data and technologies.

New materials:

- Research on biodegradable electronic components.
- Plant-based polymers.
- Reducing the use of difficult-to-recycle materials.

In the face of growing consumption and rapid technological progress, the problem of managing technological waste, including textronic waste, is becoming crucial. Textronic materials, which combine textile and electronic properties, are increasingly used in various sectors of the economy, including intelligent clothing, medicine and automotive. However, their complex structure poses a challenge to existing recycling systems. For this reason, the involvement of public institutions and the academic community in creating effective public policies regarding the recycling and management of this type of waste is becoming important.

Government institutions, international organizations and the private sector play a key role in shaping recycling policies for textronic materials. In the context of Poland, the Ministry of Climate and Environment is of particular importance, dealing with regulations regarding waste management and sustainable development strategies. The Ministry of Development and Technology is responsible for supporting innovation in the industrial sector, which is crucial for the implementation of new methods of recycling textronic materials. Another important body is the General Inspectorate for Environmental Protection, which monitors compliance with regulations regarding the disposal of technological waste.

At the European Union level, the European Commission is an important entity that shapes the strategies of the circular economy, setting standards for the recycling of textiles and electronics. The European Environment Agency provides data and analyses supporting the development of effective regulations in the field of textronic waste management.

Equally important is the role of non-governmental organizations, such as Greenpeace or the Ellen MacArthur Foundation, which not only monitor the actions of governments but also conduct information campaigns and support the development of innovative recycling technologies. The activities of these entities also support private companies that introduce innovative methods of recovering raw materials from textronic materials.

The academic community plays a key role in research on new recycling technologies and in public education on sustainable waste management. One of the priorities should be conducting research on methods of recovering raw materials from textronic materials. Modern recycling technologies are not always adapted to this type of product, which is why the development of biodegradable and recyclable textronic composites is becoming crucial.

An important aspect of academic cooperation with industry is the creation of interdisciplinary consortia that combine scientific research with the practical implementation of innovations. Universities can also support the public sector by developing analyses and reports on the effectiveness of existing regulations and recommending legislative changes.

Education and raising public awareness are equally important. Curricula should include the topic of the circular economy, considering the specificity of textronic waste. Universities can organize training and information campaigns aimed at industry and society, promoting sustainable practices in waste management.

An effective recycling policy for textronic materials requires the cooperation of many entities, including public institutions, non-governmental organizations, the private sector and the academic community. The development of regulations adapted to the specificity of textronics, the development of new recycling methods and broad-based public education are key. Integration of activities at the national and international level will allow for the creation of an effective system for managing textronic waste, contributing to environmental protection and promoting the circular economy.

## 6. Conclusions

The modern textile and electronics industry is facing a growing problem of waste resulting from the short life cycle of products and the difficulty of their reprocessing. Textronics, combining advanced electronic technologies and modern textile materials, pose additional challenges for scientists and industry related to recycling and the closed-loop economy. This paper presents a comprehensive strategy for the closed loop of textronic products, covering aspects of the ecodesign, production, use, collection, recycling and implementation of appropriate legal regulations.

The basis for effective closed loop of textronic products is ecodesign, which assumes the implementation of highly recyclable materials, elimination of harmful substances and the use of modular construction, enabling easy disassembly and separation of electronic components from textile ones. An example is smart sportswear, such as T-shirts monitoring vital signs, which should be designed in a way that allows easy separation of sensors and electronics from fabrics. Research shows that appropriate design can increase the efficiency of raw material recovery by up to 40%.

At the production stage, the key aspect is the optimization of raw material and energy consumption, which allows for reducing the carbon footprint of textronic products. Calculations show that the textile industry is responsible for 10% of global carbon dioxide emissions, which forces the implementation of resource-saving technologies. An example is the use of electronic printing technology on textiles, which reduces the amount of waste compared to traditional methods of circuit assembly.

Another important element of the strategy is the use phase of products, which should be extended through repair initiatives and reuse programs. Leasing systems for smart clothing in the medical sector, in which manufacturers provide maintenance and upgrades to the electronics, can increase the lifespan of products by 30%. Additionally, the introduction of digital product passports, containing information on the material composition and recycling instructions, allows users to make more informed decisions about the end of the product’s life cycle.

The collection and segregation process of textronic products is a major challenge due to the need to separate organic from inorganic materials. Modern identification systems, such as RFID tags or chemical markers, can support automatic waste sorting. In Scandinavian countries, the implementation of intelligent collection systems has allowed for the recovery of over 50% of materials from electronic textiles.

The final stage of the closed loop is processing and recycling, which should include both mechanical and chemical methods of recovering raw materials. Mechanical recycling allows for the recovery of textile fibers, while chemical processes enable the separation of rare metals and electronic systems.

The implementation of the closed-loop economy also requires appropriate legal regulations and support systems. Extended producer responsibility (EPR) should include both ecodesign requirements and the obligation to collect and process products at the end of their life. In the European Union, the implementation of the Textile Waste Directive planned for 2025 will be a key step in this direction.

The authors would like to point out that it is difficult to conduct a cost–benefit analysis of the proposed recycling strategies for textronic products, focusing on the economic feasibility of modular designs, recycling infrastructures, and material recovery processes, as these are relatively new material solutions. Companies are just starting to develop textronic products, and it is our role as scientists to signal the problem and emphasize the development of recyclable materials. In this article, we try to convey the message that the challenge is not the lack of technology or knowledge but rather the high costs that often make recycling economically unprofitable.

## Figures and Tables

**Table 1:** Material recovery processes.

Material Type	Recovery/Recycling Method	Reuse Example
Synthetic textiles	Mechanical shredding	Production of new fibers
Electronic components	Recovery of precious metals	Production of new devices
Biodegradable fabrics	Composting	Soil fertilization
Batteries	Chemical recycling	Recovery of lithium and other metals

**Table 2:** Comparison of textronic recycling methods and reverse logistics management.

Recycling Method	Recovery Efficiency (%)	Operating Cost (EUR/kg)	Environmental Impact	Applicability
Mechanical	60	0.5	Medium	Textile fibers
Chemical	85	1.2	High	Metals and polymers
Biological	50	0.8	Low	Biodegradable components
